# Dosimetric comparison of CT-guided iodine-125 seed stereotactic brachytherapy and stereotactic body radiation therapy in the treatment of NSCLC

**DOI:** 10.1371/journal.pone.0187390

**Published:** 2017-11-09

**Authors:** Ranran Li, Ying Zhang, Yuan Yuan, Qi Lin, Jianjian Dai, Ruicai Xu, Xudong Hu, Mingyong Han

**Affiliations:** 1 Cancer Therapy and Research Center, Shandong Provincial Hospital affiliated to Shandong University, Jinan, P. R. China; 2 Department of Radiation Oncology, Shandong Cancer Hospital affiliated to Shandong University, Jinan, P. R. China; Technische Universitat Munchen, GERMANY

## Abstract

This study aimed to assess the dosimetric differences between iodine-125 seed stereotactic brachytherapy (SBT) and stereotactic body radiation therapy (SBRT) in the treatment of non-small cell lung cancer (NSCLC). An SBT plan and an SBRT plan were generated for eleven patients with T1-2 NSCLC. Prescription of the dose and fractionation (fr) for SBRT was 48Gy/4fr. The planning aim for SBT was D90 (dose delivered to 90% of the target volume)≥120Gy. Student’s paired *t* test was used to compare the dosimetric parameters. The SBT and SBRT plans had comparable PTV D90 (104.73±2.10Gy*vs*.107.64±2.29Gy), and similar mean volume receiving 100% of the prescription dose (V100%) (91.65% *vs*.92.44%, *p* = 0.410). The mean volume receiving 150% of the prescribed dose (V150%) for SBT was 64.71%, whereas it was 0% for SBRT. Mean heterogeneity index (HI) deviation for SBT *vs*. SBRT was 0.73 *vs*. 0.19 (*p*<0.0001), and the mean conformity index (CI) for SBT *vs*. SBRT was 0.77 *vs*. 0.81 (*p* = 0.031). The mean lung doses (MLD) in SBT were significantly lower than those in SBRT (1.952±0.713 *vs*. 5.618±2.009, *p*<0.0001). In conclusion, compared with SBRT, SBT can generate a comparable dose within PTV, while the organs at risk (OARs) only receive a very low dose. But the HI and CI in SBT were lower than in SBRT.

## Introduction

Lung cancer is the leading cause of cancer death in China[1]. Surgery is usually the standard option for the treatment of localized cancers, but not all patients with operable non-small cell lung cancer (NSCLC) are candidates for surgery[2]. Stereotactic body radiation therapy (SBRT), which delivers an ablative dose of radiation over a short period of time, has emerged to become a major noninvasive option for the treatment of early stage NSCLC[3–7]. This therapy is noninvasive and well-studied, and has high local control rates. Recently, some researchers have chosen iodine-125 seeds stereotactic brachytherapy (SBT) as a treatment option for inoperable early-stage NSCLC and found that it is also a safe and effective technique[8–10]. However, there are no reports addressing the dosimetric comparison between SBT and SBRT in the treatment of early stage NSCLC.

In the present study, we assess the dosimetric differences between SBT and SBRT in the treatment of early stage NSCLC.

## Materials and methods

### Patient characteristics

Eleven consecutive patients with T1-2 NSCLC were selected for this dosimetric study. All of them had undergone SBT at Shandong Provincial Hospital affiliated to Shandong University. The patients’ characteristics are outlined in Table 1. This study was approved by the Ethics Committee of Shandong Provincial Hospital affiliated to Shandong University, Shandong, China.

**Table 1:** Patient characteristics.

No.	Gender	Age	Stage	Histology	Location
1	F	58	T1bN1M1,IV	ad	peripheral
2	M	63	T1N3M1,IV	nos	peripheral
3	F	59	T1bN3M1b,IV	ad	peripheral
4	M	66	T1N3M0,IIIB	nos	peripheral
5	F	78	T1bN2M1,IV	ad	peripheral
6	F	70	T1N0M0,IA	ad	peripheral
7	M	67	T1aN0M0,IA	ad	peripheral
8	M	62	T1N0M0,I	ad	peripheral
9	M	69	T2N3M0,IIIB	ad	peripheral
10	F	65	T2N3M1,IV	ad	peripheral
11	F	70	T1bN0M0,IA	ad	peripheral

*Abbreviations*: M = male; F = female; ad = adenocarcinoma; nos = non-small-cell lung cancer;

### Iodine-125 seed stereotactic brachytherapy

Computed tomography (CT) images were obtained under normal quiet breathing with 1.25-mm slice thickness and interval in the supine position. All CT scanning was done with *IV* contrast. The gross tumor volume (GTV) was delineated by an experienced physician on serial CT images using lung window. The clinical target volume (CTV) was generated by adding a margin of 6-8mm to GTV in all directions[11]. The planning target volume (PTV) was equal to the CTV. Contouring of the esophagus, heart, greatvessels, spinal cord, trachea plus proximal bronchial tree (central airway), and lungs minus GTV was performed in accordance with RTOG 0236 and 0813 guidelines[12,13].

SBT plans were designed using the low-dose-rate brachytherapy treatment planning system (TPS) (Prowess, version 5.0, Prowess Inc, USA). The planning aim was to give≥120 Gy to 90% of the PTV, and 100% of the PTV needed to receive at least 90% of the prescription dose[14]. All plans were generated in the preplanning module, which determined the number of needle trajectories, the number of seeds, and the total activity to be implanted. The entry site and path of the needles were chosen to avoid anatomic barriers (i.e. ribs, etc.) and damage of vital structures (i.e. large vessels, heart, etc.). In general, 3–15 interstitial needles (15-20cm long, 18-gauge) were designed to insert into the tumor at an interval of 0.5–1.0cm. The iodine-125 seeds (0.6mCi) were designed to be placed inside the CTV at an interval of 0.5–1.0 cm via needle trajectories. The median number of iodine-125seeds used was 26 (range 12–54). Based on the spatial relationship between iodine-125 seeds and the PTV, the TPS generated a dose-volume histogram (DVH) which provided dosimetric parameters such as D90 (dose delivered to 90% of the target volume) and V100% (volume receiving 100% of the prescription dose).

### Stereotactic bodyradiation therapy

SBRT plans were designed using the treatment planning system (Pinnacle, version 9.0, Philips Medical Systems, USA). CT images were transferred to the treatment planning system via disc. The GTV was delineated by the same physician with SBT. The CTV was equaled to the GTV. The PTV was defined as the CTV with a margin of 0.5cm in the axial plane and 1.0 cm in the longitudinal plane (craniocaudal). The organs at risk, including the esophagus, heart, great vessels, spinal cord, trachea plus proximal bronchial tree (central airway), and lungs minus GTV were contoured by the same physician with SBT, in accordance with the radiation therapy oncology group (RTOG) 0236 and 0813 guidelines.

The target prescription doses was 48 Gy in 4 fractions. Three-dimensional treatment planning was used to stereotactically direct a total of 10 to 12 non-coplanar, non-opposing beams to deliver the dose to the PTV. Treatment planning goals included covering at least 95% of the PTV with the prescription dose, and centering the point of maximum dose (at least 120% of the prescription dose) inside the GTV. Heterogeneity corrections were used routinely for dose calculations. DVH data was exported, and dosimetry was analyzed for the esophagus, heart/pericardium, great vessels, spinal cord, lungs minus GTV, and central airway, and compared with RTOG 0813 constraints.

### Dosimetric comparison

For each patient, DVHs, isodose distributions, and various dosimetric parameters were generated and calculated for SBT and SBRT plans, and then the dosimetric comparison of the two plans was carried out. To compare the different treatment plans, doses were converted to the biologically equivalent dose (BED) by applying the linear-quadratic model[14]. CT voxels within a delineated structure were assigned the following α/β ratio: 10 Gy for PTV, 3 Gy for lung, esophagus and heart, and 0.87Gy for spinal cord[15–17].

Eq 1 was applied to SBRT[18], whereas Eq 2 was used for SBT[14].

BED=D⋅(1+d/α/β) (1)

$$BED=\left(D\cdot \left(1+\frac{2\cdot D\cdot \beta }{\alpha \cdot \mu \cdot t}\right)\cdot \left(1-\left(\frac{1-e^{-\mu \cdot t}}{\mu \cdot t}\right)\right)\right)\cdot \frac{1}{1+\frac{2\cdot \beta }{\alpha }}$$ (2)

Where D is the total dose, d is the dose per fraction, μ is the repair rate constant (0.462 1/h), and t is the effective irradiation time related to ^125^I (T_*eff*_ = t = 171d and T_*eff*_ = 2∙t_1/2_/ln2). Tumor cell repopulation is neglected in the calculation of biologically equivalent dose.

### Treatment plan evaluation

To evaluate the quality of the plans in treating lung cancer, we calculated the heterogeneity index (HI) and conformity index (CI) on the basis of the DVHs of the PTVs. In SBT and SBRT, the CI was defined using the following equation[19,20]

$$CI=V_{PTV-REF}/V_{PTV}\cdot V_{PTV-REF}/V_{REF}$$ (3)

Where V_PTV-REF_ is the target volume covered by the prescription isodose surface, V_REF_ is all the volume covered by the prescription isodose surface; and V_PTV_ is the planning target volume.

In SBRT, we calculated the HI using the following equation[21]:

HI=D5%/D95% (4)

Where D5% and D95% represent the dose given to 5% and 95% of the PTV, respectively. The ideal HI value is 1, and it increases as the plan becomes less homogenous.

In SBT, we computed the HI using the equation below[22]:

$$HI=V_{PTV-REF}-V_{PTV-1.5REF}/V_{PTV-REF}$$ (5)

Where V_PTV-1.5REF_ is the target volume covered by the 1.5 times the prescription isodose surface. The ideal HI value is 1 and it decreases as the plan becomes less homogenous. We then compared the deviation between HI values and the ideal value of 1 for each patient (HI deviation).

For PTV, the volumes receiving 150% and 100%of the prescribed dose (V150%, V100%) and the D90 were compared between SBRT and SBT.

Lung toxicity parameters analyzed include the mean lung dose(MLD) and the percentage of normal lung receiving 5Gy(BED), 10Gy(BED), 15Gy(BED), 20Gy(BED), 25Gy(BED), 30Gy(BED), 35Gy(BED), 40Gy(BED), 45Gy(BED) and 50Gy(BED), (V_5Gy(BED)_, V_10Gy(BED)_, V_15Gy(BED)_, V_20Gy(BED)_, V_25Gy(BED)_, V_30Gy BED)_, V_35Gy(BED)_, V_40Gy(BED)_, V_45Gy(BED)_ and V_50Gy(BED)_ respectively). Normal lung volume was defined as the bilateral lung volume. Maximum and mean doses tospinal cord, esophagus and heart were also evaluated.

### Statistical analysis

In this study, we used SPSS 20.0 (IBM Inc, USA) and the paired *t* test for the comparison of the two groups. Experimental data are expressed as the means±standard deviation (SD), and *p*<0.05 was considered statistically significant.

## Results

### Dosimetric parameters comparison between SBT and SBRT

**Fig 1:**
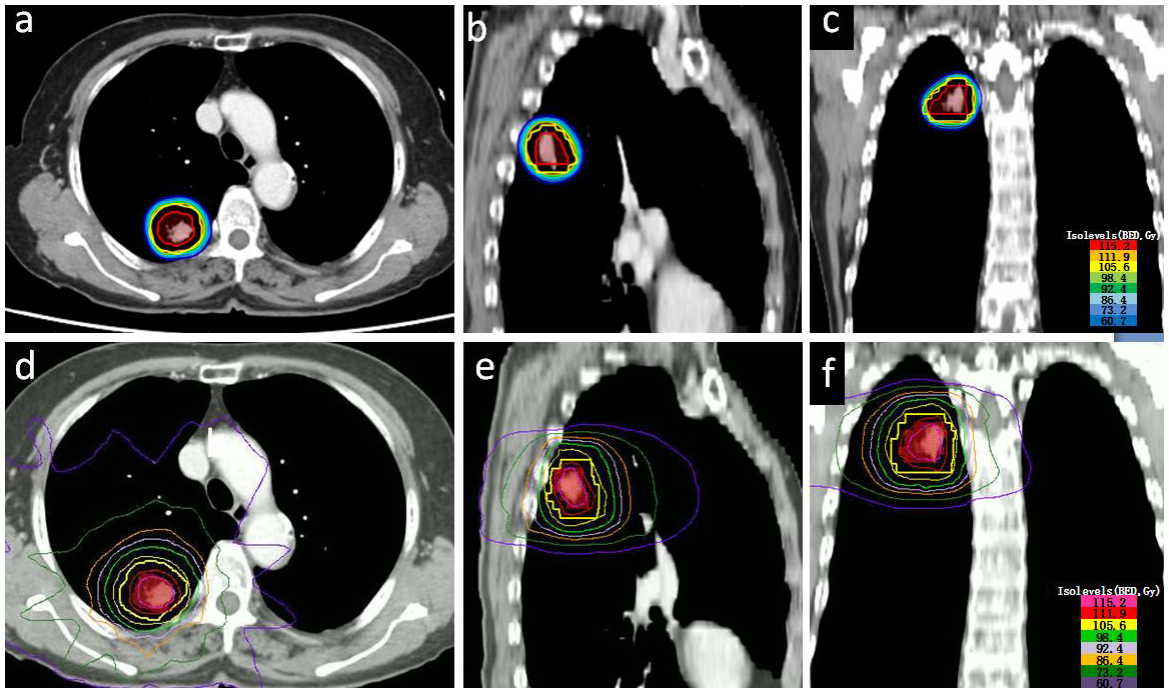
Representative dose distributions for the patient 5inSBT and SBRT after radiobiological conversion. a, b and c are the axial, sagittal and coronal dose distribution in SBT respectively. The red, orange, yellow, green-yellow, green, light sky blue, dodger blue, and corn flower blue lines represent the 115.2Gy(BED), 111.9Gy(BED), 105.6Gy(BED), 98.4Gy(BED), 92.4Gy(BED), 86.4Gy(BED), 73.2Gy(BED), and 60.7Gy(BED) isodose curves respectively. d, e and fare the axial, sagittal, and coronal dose distributions in SBRT, respectively. The hot pink, crimson, yellow, green-yellow, plum, orange, leaf green, and dark violet lines represent the 126Gy(BED), 118Gy(BED), 101Gy(BED), 92Gy(BED), 81Gy(BED), 70Gy(BED), 46Gy(BED) and 23Gy(BED) isodose curves respectively.

Prescription doses for SBRT and SBT were 48Gy and 120Gy respectively. To compare different treatment plans, doses were converted to the BED with an α/β ratio of 10Gy for PTV. Table 2 lists a summary of dosimetric parameters for PTV in the two treatment plans. In terms of target coverage, the PTV D90 of SBRT was slightly higher than that of SBT (104.73±2.10Gy *vs*.107.64±2.29Gy, *p* = 0.006), and the mean difference was 2.9Gy. SBT and SBRT plans had similar PTV V100% (91.65±1.06% *vs*. 92.44±2.62%, *p* = 0.410), whereas the PTV V150% for SBT and SBRT was 64.71±5.8% *vs*. 0±0%, indicating significant dose escalation within the PTV with SBT. We then compared the deviation between HI values and the ideal value of 1 for each treatment plan (HI deviation). Mean HI deviation of the PTV for SBT *vs*. SBRT was 0.73±0.06*vs*. 0.19±0.051, respectively (*p*<0.001). The CI for PTV of SBT*vs*. SBRT was0.77±0.05 *vs*. 0.81±0.06, respectively (*p* = 0.031). Fig 1 depicts the dose distributions in the transverse, coronal and sagittal planes in SBT and SBRT plans.

**Table 2:** Dosimetric parameters comparison between SBT and SBRT.

Dosimetric index	SBT(means±SD)	SBRT(means±SD)	*p*
Prescription dose(Gy)	120	48	-
Prescription dose(BED)(Gy)	100.86	105.6	-
D90(Gy)	124.70±2.91	48.87±1.01	-
D90(BED)(Gy)	104.73±2.10	107.64±2.29	0.006
V150 (%)	64.71±5.80	0±0	-
V100 (%)	91.65±1.06	92.44±2.62	0.410
V95 (%)	94.13±2.12	99.25±0.64	<0.001
HI	0.27±0.06	1.19±0.051	-
HI deviation	0.73±0.06	0.19±0.051	<0.001
CI	0.77±0.05	0.81±0.06	0.031

### Dosimetric comparison of the whole lung between SBT and SBRT

**Fig 2:**
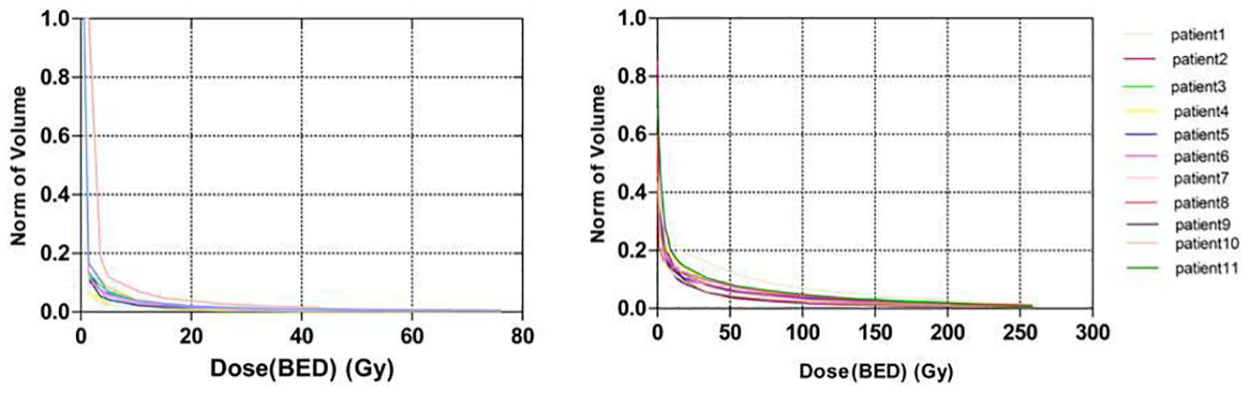
Dose-volume histogram of the lung for eleven patients after radiobiological conversion in SBT and SBRT plans.

The percentages of total (whole) lung volume receiving 5Gy(BED), 10Gy(BED), 15Gy(BED), 20Gy(BED), 25Gy(BED), 30Gy(BED), 35Gy(BED), 40Gy(BED), 45Gy(BED), and 50Gy(BED) were all significantly lower in the SBT group compared with the SBRT group (Fig 2, Table 3). The MLD in the SBT group were significantly lower than those in the SBRT group (1.95±0.71Gy *vs*. 20.56±5.65Gy, *p*<0.0001) (Table 3).

**Table 3:** Dosimetric comparison of the whole lung between SBT and SBRT.

Parameters	SBT (means±SD)	SBRT (means±SD)	*p*
V5	5.854±2.730%	21.214±5.523%	<0.0001
V10	3.372±1.655%	16.350±3.713%	<0.0001
V15	2.391±1.064%	12.748±3.305%	<0.0001
V20	1.754±0.846%	11.788±3.262%	<0.0001
V25	1.945±1.785%	10.243±3.261%	<0.0001
V30	1.109±0.516%	9.615±3.245%	<0.0001
V35	0.945±0.445%	8.531±3.077%	<0.0001
V40	0.791±0.388%	7.978±2.901%	<0.0001
V45	0.645±0.266%	7.044±2.628%	<0.0001
V50	0.563±0.220%	12.209±2.971%	<0.0001
MLD	1.952±0.713Gy	5.618±2.009Gy	<0.0001

### Dosimetric comparison of the organs at risk (OARs) between SBT and SBRT

For OARs, the Dmax for the spinal cord (0.840±1.605Gy *vs*. 62.081±86.306Gy, *p* = 0.038), esophagus (0.855±1.032Gy *vs*. 21.521±14.167Gy, *p*<0.0001) and heart (1.981±3.365 *vs*.50.004±69.080Gy, *p* = 0.040) in SBT were significantly lower than those in SBRT. (Table 4)

**Table 4:** Dosimetric comparison of the OARs between SBT and SBRT after radiobiological conversion.

Organs	Parameters	SBT(means±SD)Gy	SBRT(means±SD)Gy	*p*
Spinal cord	Dmax	0.840±1.605	62.081±86.306	0.038
	Dmean	0.252±0.482	5.170±5.778	0.016
	Dmin	0.002±0.003	0.049±0.038	0.002
Heart	Dmax	1.981±3.365	50.004±69.080	0.040
	Dmean	0.690±0.120	2.582±2.593	0.034
	Dmin	0.015±0.088	0.013±0.019	0.002
Esophagus	Dmax	0.855±1.032	21.521±14.167	<0.0001
	Dmean	0.705±0.185	3.300±2.253	0.002
	Dmin	0.016±0.015	0.072±0.038	<0.0001

## Discussion

SBRT has evolved to become a standard option for the treatment of inoperable early stage NSCLC. It is noninvasive and mature, and most of the existing literature uses this technique[3–7]. Recently, some researchers reported that SBT is also a safe and effective modality for the treatment of inoperable early stage NSCLC[8–10]. Pennington JD et al. compared the dosimetric differences between brachytherapy and SBRT in the treatment of liver metastasis[23]. In their study, brachytherapy led to a higher target dose, a similar dose to OARs, but potentially had lower target coverage compared with SBRT. Milickovic Net al. compared the dosimetric differences between interstitial Ir-192 high-dose-rate (HDR) brachytherapy and linear accelerator-based SBRT for intrathoracic malignancies[24]. They showed that volume coverage in HDR plans was comparable to that calculated for SBRT with no statistically significant difference in terms of conformity. In addition, they found that the dose falloff gradient-sharpness of the HDR plans was considerably steeper compared with the SBRT plans. Now, we also conducted a treatment planning study to compare the dosimetric differences between SBT and SBRT in the treatment of NSCLC.

In our study, the iodine-125 seeds were implanted into tumor volume under CT guidance. We used the low-dose-rate brachytherapy planning software (Prowess 5.0) to create a treatment plan for each patient. The treatment was typically delivered in a single fraction; the PTV did not need to account for the motion of the CTV and setup deviations. Therefore, the PTV was equal to the CTV in SBT treatment planning. Because of the smaller PTV, we speculated that the radiation injury to nearby organs in SBT may be lower than that in SBRT.

The overall dose delivered to the tumor is strongly associated with local control. For SBRT, several studies suggested that a BED of ≥100 Gy delivered to the periphery of the tumor is ideal[25]. The BED is a characteristic dose value that facilitates comparisons among different dose-fractionation schemes. In our research, the SBT and SBRT plan had comparable PTV D90 values (104.73±2.10Gy *vs*. 107.64±2.29Gy), which is in agreement with the results of Pennington J Det al.[23] and Milickovic N et al.[24].

With comparable BED values, we compared the CI, HI, V100% and V150% in terms of PTV. We found similar V100% values for SBT and SBRT plans, but the V150% values of SBT plans were significantly higher than those of SBRT plans, meaning the SBT plans were less homogeneous than the SBRT plan. We then compared the deviation between HI values and the ideal value of 1 for each treatment planning. The HI deviations in SBT were much higher than in SBRT (*p*<0.001), indicating that SBT generates highly in homogeneous spatial dose distributions across the PTV. Previous studies proposed that tissue heterogeneity and interseed attenuationare somehow contributing to the heterogeneity of dose distribution[26]. The heterogeneous dose distribution may create a higher likelihood of unintended low-dose areas, leading to a higher rate of tumor recurrence.

The CI, which represents the relationship among isodose distributions, target volume and healthy tissue, is very useful in assessing the quality of radiotherapy treatment plans[27]. This index has a value between 0 and 1. A value of 1 implies high planning target volume coverage and minimal unnecessary irradiation of surrounding tissues. van't Riet A et al. suggested that treatment with a CI above 0.60 indicates optimal treatment planning[20,28]. In our study, the CI for PTV of SBT and SBRT were 0.77±0.05 and 0.81±0.06, respectively (*p* = 0.031). This finding indicates that, compared with SBT, SBRT provides better conformal dose distribution in the treatment of NSCLC.

Radiation pneumonitis (RP) is the major toxicity that limits the radiation dose that can be delivered in the treatment of lung cancers[17]. Previous studies demonstrated that the MLD, V5 and V20 were correlated with the incidence of RP[7]. In this study, all of these dosimetric factors were significantly lower in SBT compared with SBRT. The vicinity of OARs, including the spinal cord, heart and esophagus, also limits the delivery of effective radiation doses in the treatment of thoracic cancers. Our data showed that SBT demonstrated a reduced dose to adjacent critical structures compared with SBRT.

What is the best radiation therapy treatment technique for inoperable early stage NSCLC? Although our dosimetric assessment demonstrated that SBT was superior to SBRT, other factors might have an impact on the decision making process. In SBT, we implanted iodine-125 seeds into lung tumors percutaneously. In contrast to SBRT, SBT is an invasive technique, and the most common complications are pneumothorax and bleeding[29]. Additionally, the treatment plan must be frequently modified during the procedure, due to respiratory movement, rib blocking and the need to avoid vascular structures. Although a special effort has been made to adhere as closely as possible to the treatment plan, we have to recognize that the 100% accurate placement of seeds according to the treatment plan is usually impossible.

There are several limitations in this study. In SBRT treatment, accurate assessment of tumor dimension and range of motion is essential for disease control and protection of normal organs. Four-dimensional (4D) CT simulation is the preferred technique for assessment of tumor position and respiratory motion. However, 4DCT scanners were not available in our hospital. As a result, the influence of respiratory movements could not be evaluated in the present study. Visible GTVs on CT images were contoured to reflect the internal target volumes (ITVs).

## Conclusion

Compared with SBRT, SBT can generate a comparable dose within the PTV, while the OARs only receive a very low dose and are subject to little damage. However, the HI and CI values in SBT were lower than those in SBRT.

## Supporting information

S1 Table Doses to PTV and OARs. (XLSX)

## References

[1] Chen W, Zheng R, Baade PD, Zhang S, Zeng H, Bray F, et al Cancer statistics in China, 2015. CA Cancer J Clin. 2016 Mar-Apr; 66(2):115–32. https://doi.org/10.3322/caac.21338. 26808342 10.3322/caac.21338

[2] McGarry RC, Song G, des Rosiers P, Timmerman R. Observation-only management of early stage, medically inoperable lung cancer: poor outcome. Chest. 2002 4; 121(4):1155–8. 11948046 10.1378/chest.121.4.1155

[3] Falkson CB, Vella ET, Yu E, El-Mallah M, Mackenzie R, Ellis PM, et al Guideline for radiotherapy with curative intent in patients with early-stage medically inoperable non-small-cell lung cancer. Curr Oncol. 2017 2; 24(1):e44–e49. https://doi.org/10.3747/co.24.3358 28270731 10.3747/co.24.3358 PMC5330637

[4] Roach MC, Videtic GM, Bradley JD. Treatment of Peripheral Non-Small Cell Lung Carcinoma with Stereotactic Body Radiation Therapy. J Thorac Oncol. 2015 9; 10(9):1261–67. https://doi.org/10.1097/JTO.0000000000000610 26291009 10.1097/JTO.0000000000000610

[5] Sapkaroski D, Osborne C, Knight KA. A review of stereotactic body radiotherapy -is volumetric modulated arc therapy the answer? J Med Radiat Sci. 2015 6; 62(2):142–51. https://doi.org/10.1002/jmrs.108 26229679 10.1002/jmrs.108 PMC4462986

[6] Tsang MW. Stereotactic body radiotherapy: current strategies and future development. J Thorac Dis. 2016 7; 8(Suppl 6):S517–27. https://doi.org/10.21037/jtd.2016.03.14 27606082 10.21037/jtd.2016.03.14 PMC4990666

[7] Kang KH, Okoye CC, Patel RB, Siva S, Biswas T, Ellis RJ, et al Complications from Stereotactic Body Radiotherapy for Lung Cancer. Cancers. 2015 6; 7(2):981–1004. https://doi.org/10.3390/cancers7020820 26083933 10.3390/cancers7020820 PMC4491695

[8] Martinez-Monge R, Pagola M, Vivas I, Lopez-Picazo JM. CT-guided permanent brachytherapy for patients with medically inoperable early-stage non-small cell lung cancer (NSCLC). Lung cancer. 2008 8; 61(2):209–13. https://doi.org/10.1016/j.lungcan doi: 10.1016/j.lungcan.2007.12.016 18243409 10.1016/j.lungcan.2007.12.016

[9] Wang ZM, Lu J, Liu T, Chen KM, Huang G, Liu FJ. CT-guided interstitial brachytherapy of inoperable non-small cell lung cancer. Lung cancer. 2011 11; 74(2):253–7. https://doi.org/10.1016/j.lungcan.2011.03.006 21513997 10.1016/j.lungcan.2011.03.006

[10] Li J, Yu M, Xiao Y, Yang L, Zhang J, Ray E, et al Computed tomography fluoroscopy-guided percutaneous 125I seed implantation for safe, effective and real-time monitoring radiotherapy of inoperable stage T1-3N0M0 non-small-cell lung cancer. Mol Clin Oncol. 2013 11; 1(6):1019–24. doi: 10.3892/mco.2013.171 24649287 10.3892/mco.2013.171 PMC3915541

[11] Giraud P, Antoine M, Larrouy A, Milleron B, Callard P, De Rycke Y, et al Evaluation of microscopic tumor extension in non-small-cell lung cancer for three-dimensional conformal radiotherapy planning. Int J Radiat Oncol Bio Phys. 2000 11; 48(4):1015–24. 11072158 10.1016/s0360-3016(00)00750-1

[12] Michalski J, Timmerman R, Fowler J, Gore E, Timmerman RD, Choy H, et al. A phase II trial of SBRT in the treatment of patients with medicallyinoperable stage I/II NSCLC. RTOG 0236. RTOG;2009:1–39.

[13] Bezjak A, Bradley J, Gaspar L, Robert D, Papiez L, Gore E, et al.Seamless phase I/II study of stereotactic lung radiotherapy(SBRT) for early stage,centrally located, non-small-cell lung cancer(NSCLC) in medically inoperable patients. RTOG 0813. RTOG;2015:1–75.

[14] Georg D, Hopfgartner J, Gora J, Kuess P, Kragl G, Berger D, et al Dosimetric considerations to determine the optimal technique for localized prostate cancer among external photon, proton, or carbon-ion therapy and high-dose-rate or low-dose-rate brachytherapy. Int J Radiat Oncol Bio Phys. 2014 3; 88(3):715–22. https://doi.org/10.1016/j.ijrobp.2013.11.241 24521685 10.1016/j.ijrobp.2013.11.241

[15] Chi A, Wen S, Liao Z, Fowler J, Xu J, Nguyen NP, et al What would be the most appropriate alpha/beta ratio in the setting of stereotactic body radiation therapy for early stage non-small cell lung cancer. Biomed Res Int. 2013; 2013:391021 https://doi.org/10.1155/2013/391021 24350266 10.1155/2013/391021 PMC3853037

[16] Schultheiss TE. The radiation dose-response of the human spinal cord. Int J RadiatOncol Bio Phys. 2008 8; 71(5):1455–9. https://doi.org/10.1016/j.ijrobp.2007.11.075 18243570 10.1016/j.ijrobp.2007.11.075

[17] Borst GR, Ishikawa M, Nijkamp J, Hauptmann M, Shirato H, Bengua G, et al Radiation pneumonitis after hypofractionated radiotherapy: evaluation of the LQ(L) model and different dose parameters. Int J Radiat Oncol Bio Phys. 2010 8; 77(5):1596–603. https://doi.org/10.1016/j.ijrobp.2009.10.015 20231066 10.1016/j.ijrobp.2009.10.015

[18] Davis JN, Medbery C, Sharma S, Pablo J, Kimsey F, Perry D, et al Stereotactic body radiotherapy for centrally located early-stage non-small cell lung cancer or lung metastases from the RSSearch((R)) patient registry. Radiat Oncol. 2015 5;10:113 https://doi.org/10.1186/s13014-015-0417-5 25975848 10.1186/s13014-015-0417-5 PMC4443630

[19] Feuvret L, Noel G, Mazeron JJ, Bey P. Conformity index: a review. Int J Radiat Oncol Bio Phys. 2006 2; 64(2):333–42. doi: 10.1016/j.ijrobp.2005.09.028 16414369 10.1016/j.ijrobp.2005.09.028

[20] van't Riet A, Mak AC, Moerland MA, Elders LH, van der Zee W. A conformation number to quantify the degree of conformality in brachytherapy and external beam irradiation: application to the prostate. Int J Radiat Oncol Bio Phys. 1997 2;37(3):731–6. 9112473 10.1016/s0360-3016(96)00601-3

[21] Kataria T, Sharma K, Subramani V, Karrthick KP, Bisht SS. Homogeneity Index: An objective tool for assessment of conformal radiation treatments. J Med Phys. 2012 10; 37(4):207–13. https://doi.org/10.4103/0971-6203.103606 23293452 10.4103/0971-6203.103606 PMC3532749

[22] Saw CB, Suntharalingam N. Quantitative assessment of interstitial implants. Int J Radiat Oncol Bio Phys. 1991 1; 20(1):135–9. 1993622 10.1016/0360-3016(91)90149-x

[23] Pennington JD, Park SJ, Abgaryan N, Banerjee R, Lee PP, Loh C, et al Dosimetric comparison of brachyablation and stereotactic ablative body radiotherapy in the treatment of liver metastasis. Brachytherapy. 2015 Jul-Aug; 14(4):537–42. https://doi.org/10.1016/j.brachy.2015.04.002 25944395 10.1016/j.brachy.2015.04.002

[24] Milickovic N, Tselis N, Karagiannis E, Ferentinos K, Zamboglou N. Iridium-Knife: Another knife in radiation oncology. Brachytherapy. 2017 Jul-Aug; 16(4):884–92. https://doi.org/10.1016/j.brachy.2017.03.005 28392144 10.1016/j.brachy.2017.03.005

[25] Loganadane G, Martinetti F, Mercier O, Krhili S, Riet FG, Mbagui R, et al Stereotactic ablative radiotherapy for early stage non-small cell lung cancer: A critical literature review of predictive factors of relapse. Cancer Treat Rev. 2016 11; 50:240–6. https://doi.org/10.1016/j.ctrv.2016.10 doi: 10.1016/j.ctrv.2016.10.002 27768919 10.1016/j.ctrv.2016.10.002

[26] Afsharpour H, Reniers B, Landry G, Pignol JP, Keller BM, Verhaegen F, et al Consequences of dose heterogeneity on the biological efficiency of (1)(0)(3)Pd permanent breast seed implants. Phys Med and Biol. 2012 2;57(3):809–23. https://doi.org/10.1088/0031-9155/57/3/80922252246 22252246 10.1088/0031-9155/57/3/809

[27] Brennan SM, Thirion P, Buckney S, Shea CO, Armstrong J. Factors influencing conformity index in radiotherapy for non-small cell lung cancer. Med Dosim. 2010 Spring; 35(1):38–42. https://doi.org/10.1016/j.meddos.2009.01.003 19931013 10.1016/j.meddos.2009.01.003

[28] Giglioli FR, Strigari L, Ragona R, Borzi GR, Cagni E, Carbonini C, et al Lung stereotactic ablative body radiotherapy: A large scale multi-institutional planning comparison for interpreting results of multi-institutional studies. Physic Med 2016 4; 32(4):600–6. https://doi.org/10.1016/j.ejmp.2016.03.015 27061871 10.1016/j.ejmp.2016.03.015

[29] Heelan RT, Hilaris BS, Anderson LL, Nori D, Martini N, Watson RC, et al Lung tumors: percutaneous implantation of I-125 sources with CT treatment planning. Radiology. 1987 9;164(3):735–40. doi: 10.1148/radiology.164.3.3615870 3615870 10.1148/radiology.164.3.3615870

